# Validation of the European Laryngological Society classification of glottic vascular changes as seen by narrow band imaging in the optical biopsy setting

**DOI:** 10.1007/s00405-021-06723-7

**Published:** 2021-03-12

**Authors:** Francesco Missale, Stefano Taboni, Andrea Luigi Camillo Carobbio, Francesco Mazzola, Giulia Berretti, Andrea Iandelli, Marco Fragale, Francesco Mora, Alberto Paderno, Francesca Del Bon, Giampiero Parrinello, Alberto Deganello, Cesare Piazza, Giorgio Peretti

**Affiliations:** 1 IRCCS Ospedale Policlinico San Martino, Genoa, Italy; 2 Department of Molecular and Translational Medicine, University of Brescia, Brescia, Italy; 3 Section of Otorhinolaryngology, Head and Neck Surgery, Azienda Ospedaliera di Padova, University of Padua, Padua, Italy; 4 Department of Clinical and Experimental Sciences, University of Brescia, Brescia, Italy; 5 Department of Surgical Sciences and Integrated Diagnostics (DISC), University of Genoa, Genoa, Italy; 6 Department of Otolaryngology, Head and Neck Surgery, IRCCS Regina Elena National Cancer Institute, Rome, Italy; 7 Unit of Otorhinolaryngology, Head and Neck Surgery, ASST Spedali Civili di Brescia, University of Brescia, Brescia, Italy

**Keywords:** Narrow band imaging, European Laryngological Society classification, Optical biopsy, Laryngeal cancer, Vascular changes, Endoscopy

## Abstract

**Purpose:**

In 2016, the European Laryngological Society (ELS) proposed a classification for vascular changes occurring in glottic lesions as visible by narrow band imaging (NBI), based on the dichotomic distinction between longitudinal vessels (not suspicious) and perpendicular ones (suspicious). The aim of our study was to validate this classification assessing the interobserver agreement and diagnostic test performance in detecting the final histopathology.

**Methods:**

A retrospective study was carried out by reviewing clinical charts, preoperative videos, and final pathologic diagnosis of patients submitted to transoral microsurgery for laryngeal lesions in two Italian referral centers. In each institution, two physicians, independently re-assessed each case applying the ELS classification.

**Results:**

The cohort was composed of 707 patients. The pathologic report showed benign lesions in 208 (29.5%) cases, papillomatosis in 34 (4.8%), squamous intraepithelial neoplasia (SIN) up to carcinoma in situ in 200 (28.2%), and squamous cell carcinoma (SCC) in 265 (37.5%). The interobserver agreement was extremely high in both institutions (*k* = 0.954, *p* < 0.001 and *k* = 0.880, *p* < 0.001). Considering the diagnostic performance for identification of at least SIN or SCC, the sensitivity was 0.804 and 0.902, the specificity 0.793 and 0.581, the positive predictive value 0.882 and 0.564, and the negative predictive value 0.678 and 0.908, respectively.

**Conclusion:**

The ELS classification for NBI vascular changes of glottic lesions is a highly reliable tool whose systematic use allows a better diagnostic evaluation of suspicious laryngeal lesions, reliably distinguishing benign ones from those with a diagnosis of papillomatosis, SIN or SCC, thus paving the way towards confirmation of the optical biopsy concept.

## Introduction

Early detection and diagnosis of laryngeal squamous cell carcinoma (SCC) are crucially involved not only in reducing mortality, but also to optimize therapeutic approaches aimed at achieving the best organ and functional preservation [1, 2]. Fortunately, glottic SCC, the most common laryngeal tumor localization, is more frequently detected at an earlier stage than tumors originating in other subsites of the head and neck due to its early (albeit highly non-specific) symptoms [3]. Laryngeal SCC examination is usually performed by flexible (video)endoscopy under white light (WL) and relies on the analysis of superficial characteristics (size, color, location, single or multifocal appearance) and visible morphological features (smoothness, irregularity, keratinization, ulceration, submucosal growth), per se non-pathognomonic and possibly overlapping each other in malignant and benign pathologies, especially when diagnosed at early stages. This implies the frequent need to obtain an incisional biopsy before deciding on the therapeutic approach, with an increase in costs, anesthetic risks, and potential undue damage to the vocal cords.

Computed tomography and magnetic resonance imaging definitively play a major role in diagnosis of more advanced diseases, providing information about the involvement of laryngeal structures and lateral neck lymph nodes, while, on the other hand, they fall shortly in identifying and characterizing superficial mucosal lesions. By contrast, the judicious use of high definition and better contrasted videoendoscopic images now offer staggering details in evaluation of epithelial and superficial vascular patterns. Moreover, clinicians can increasingly benefit from novel optical diagnostic methods, providing information even closer to those obtained by formal histopathological examination, thus differentiating between normal mucosa and discrete lesions and, among the latter, between those with benign versus malignant behaviors [4]. In this context, narrow band imaging (NBI) is a well-established bioendoscopic technique using filtered wavelengths to enhance microvascular alterations associated with preneoplastic and neoplastic transformation of the upper aerodigestive tract (UADT) mucosa [5–8]. Since its first introduction in the late 1990s, the use of NBI has considerably upgraded physicians’ ability for non-invasive detection and delineation of suspicious mucosal lesions, and is thus beneficial in the diagnosis of a variety of benign and malignant lesions [9]. However, the need for a common language to be shared among clinicians to describe NBI-enhanced vascular patterns led to the proposal of different classifications during the last decades [10–12]. In 2016, the Working Committee on Endoscopic Laryngeal Imaging of the European Laryngological Society (ELS) published a new proposal for a simplified (dichotomic) description of vocal fold vascular changes as seen under NBI [12]. In this system, the authors distinguished between normal and pathologic vascular patterns of the vocal folds. The latter, in turn, were divided into longitudinal and perpendicular vascular changes. Longitudinal vessels characterize benign lesions, while perpendicular ones (i.e*.* dot-like intrapapillary capillary loops [IPCL], enlarged and worm-like vessels) are considered signs of benign neoplasms (such as papillomatosis), squamous intraepithelial neoplasia (SIN), or frankly malignant lesions.

The present study aims to assess the performance of the ELS classification of vascular changes in a broad multicenter cohort, testing its interobserver agreement as primary endpoint, and analyzing its accuracy in predicting the final pathological results in an optical biopsy setting, i.e*.* by evaluating the diagnostic accuracy of NBI by comparing it with the final histopathologic diagnosis obtained after complete removal of the glottic lesion.

## Methods

### Study population

A retrospective study was carried out enrolling patients treated at the Departments of Otorhinolaryngology—Head and Neck Surgery of the Universities of Genoa (Center A; from January 2012 to December 2016) and Brescia (Center B; from January 2015 to December 2018), Italy.

All patients enrolled were affected by laryngeal lesions; a pre-treatment videoendoscopic evaluation with both WL and NBI was performed in the office as well as in the operatory theater, and the records were saved in his/her medical chart; the laryngeal lesion was treated by a transoral microsurgical approach by either cold instrumentation and carbon dioxide (CO_2_) laser; postoperative histopathologic assessment was obtained to classify the resected tissue as benign, dysplastic or malignant. Histopathological diagnosis was performed according to the WHO classification system [13].

### Clinical diagnostic work-up

All patients were preoperatively evaluated by high-definition television (HDTV)-WL and HDTV-NBI through a videorhinolaryngoscope ENF-VQ or ENF-VH coupled to an Evis Exera II HDTV camera connected to an Evis Exera II CLV-180B light source (Olympus Medical System Corp., Tokyo, Japan). Just before surgery, in the operating room, with patient under general anesthesia, intraoperative HDTV-WL and HDTV-NBI rigid endoscopy with 0° and 70° telescopes (Karl Storz, Tuttlingen, Germany) was also systematically performed. On the basis of this diagnostic work-up, laryngeal lesions were subsequently removed by either a phonomicrosurgical approach (in case of benign lesions) or excisional biopsy (in case of papillomatosis, SIN, carcinoma in situ [CIS] or invasive SCC) by type I–III cordectomies according to the ELS classification of cordectomies [14].

### Clinical evaluation applying the ELS classification

**Fig. 1:**
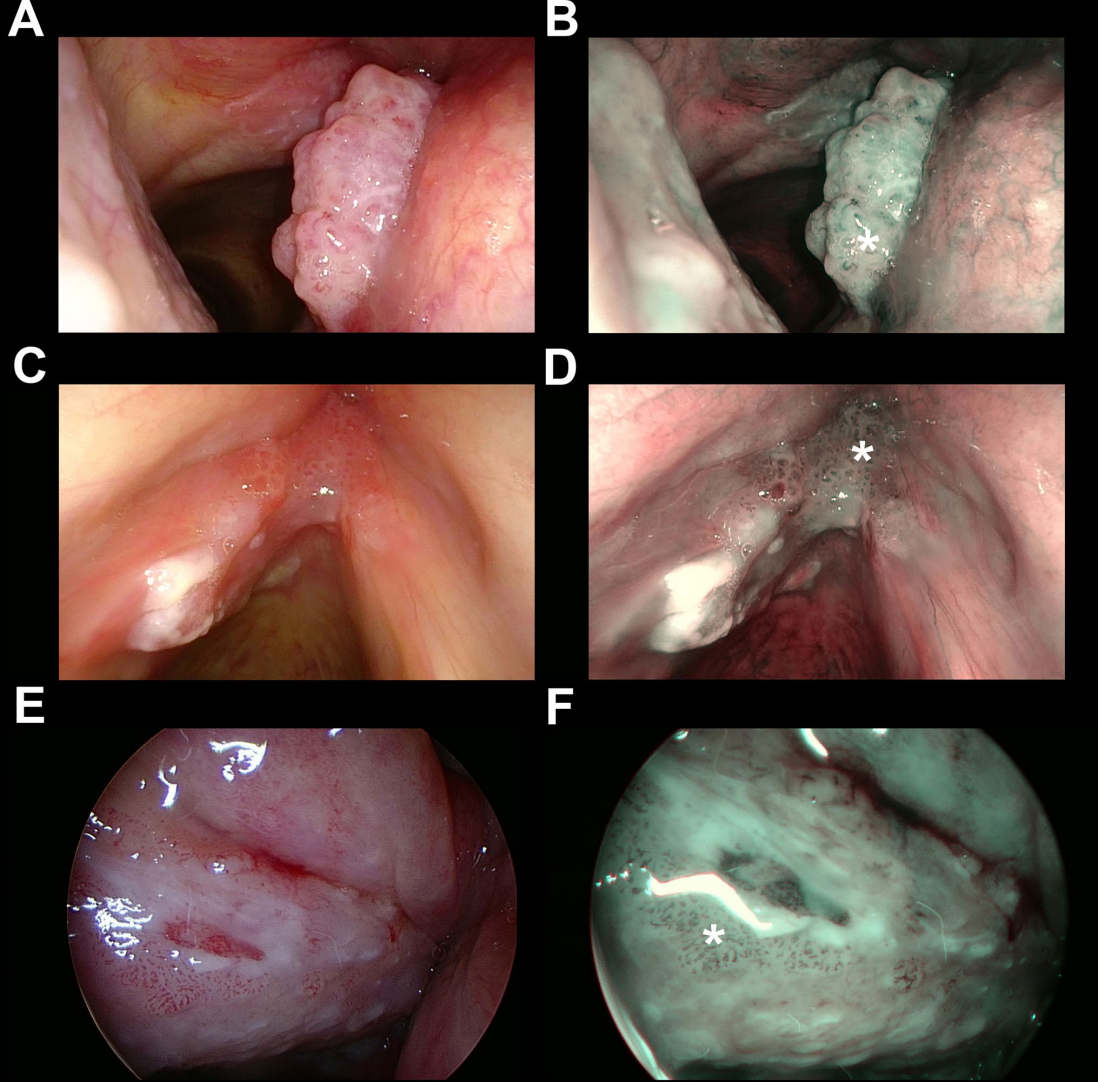
Endoscopic picture of three representative cases of SCC (**a**–**d**) or CIS (**e**, **f**) correctly identified as suspicious by the presence of perpendicular vascular abnormalities (* in all panels) evaluating the NBI endoscopic appearance (**b**, **d**, **f**) and applying the ELS classification

**Fig. 2:**
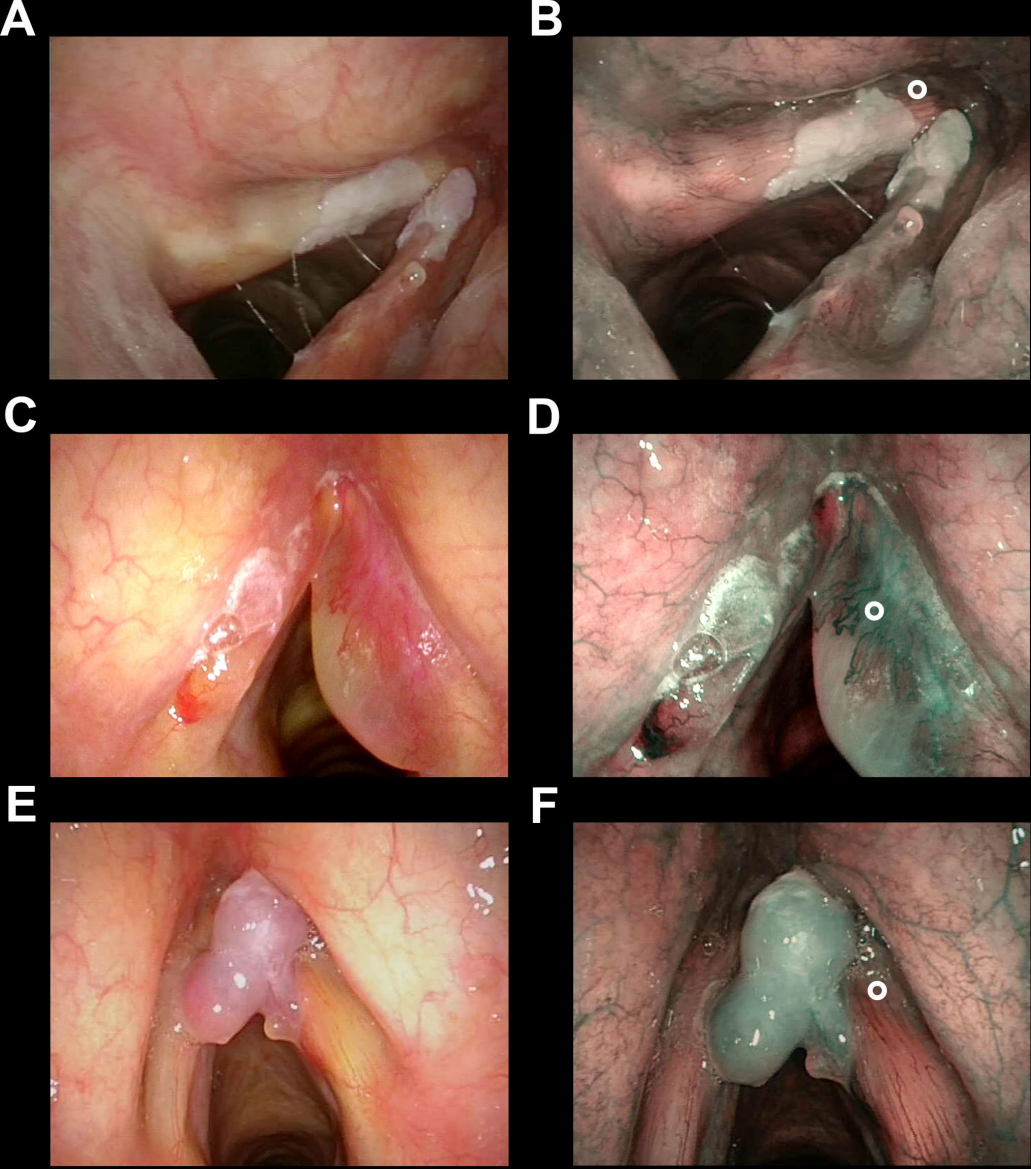
Endoscopic picture of three representative cases of benign glottic lesions: keratosis without atypia (**a**, **b**), Reinke’s edema (**c**, **d**) and polyp (**e**, **f**) correctly identified as benign lesions without identifying any perpendicular vascular abnormalities evaluating the NBI endoscopic appearance (**b**, **d**, **f**) and applying the ELS classification. The ° in all panels points to non-suspicious longitudinal vascular abnormalities that can be observed inside the lesion (**d**) of at its boundary (**b**, **f**)

**Fig. 3:**
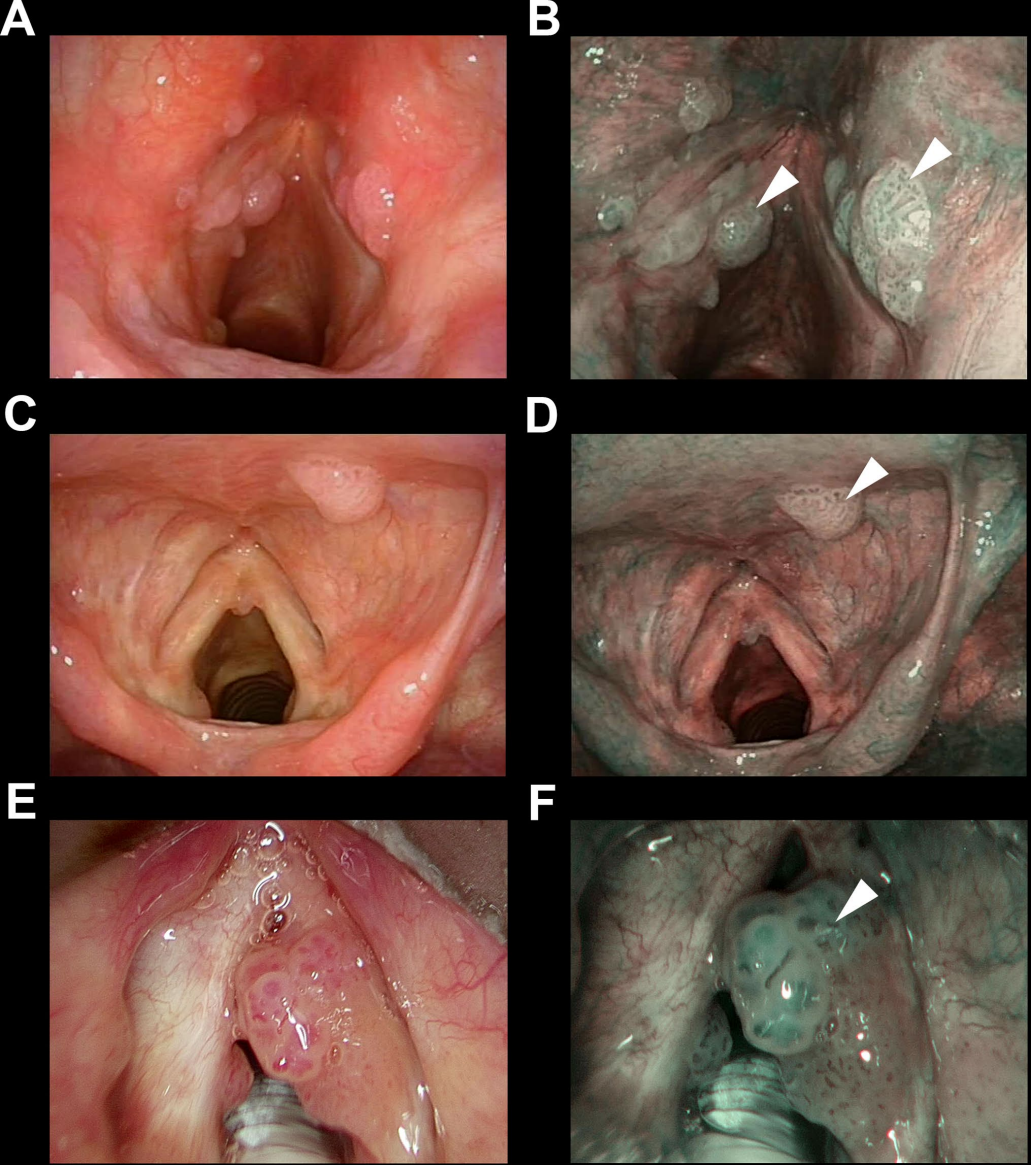
Endoscopic pictures of three representative cases (**a**, **b**; **c**, **d**; and **e**, **f**) of recurrent laryngeal papillomatosis correctly identified detecting wide angle IPCLs (arrowheads in all panels) evaluating the NBI endoscopic appearance (**b**, **d**, **f**)

Clinical records of the study population, including demographic features and information on previous treatments in terms of laryngeal surgery, head and neck radiotherapy, or other treatments before the index transoral microsurgical procedure were retrieved from the hospital databases. Two independent physicians from each institution with at least a 3-year-experience in the use of NBI, blinded to the final histopathologic result, retrospectively and independently reviewed the intraoperative videoendoscopic recordings. Applying the ELS classification for laryngeal vascular changes [15], each case was categorized as suspicious for malignancy (presence of perpendicular vascular abnormalities as shown in Fig. 1) or non-suspicious (undetectable perpendicular vascular changes or longitudinal ones as shown in Fig. 2). In case of interobserver disagreement, consensus was reached by direct comparison between the examiners. The identification of features of respiratory papillomatosis (i.e., wide angle IPCL) was also considered as a secondary endpoint (Fig. 3).

### Statistical analysis

Clinical data were reported as absolute and relative frequencies. The reliability of the ELS classification was assessed for each independent cohort measuring the Cohen’s k statistic and the strength of agreement graded according to Altman et al. [16, 17], as reported in Table 1. Considering the final consensus of the evaluation, we assessed the performance of the diagnostic test for prediction of the final pathologic result (at least SIN1, up to SCC) in an optical biopsy setting. For better understanding of the clinical utility of applying the classification and detecting papillomatosis-like features, the Clinical Utility (CU) indexes were also derived, taking into account the measures of occurrence (sensitivity or specificity) together with the possibility of discrimination (positive [PPV] or negative predictive values [NPV]), and their qualitative grading were judged accordingly [18, 19] (Table 1). The Positive Clinical Utility Index (CU + Ve) is defined as sensitivity*PPV, and a high CU + Ve results should characterize “case finding” tests. By contrast, good Negative Clinical Utility Index (CU−Ve), defined as specificity*NPV, should be ideal for “screening” tests [18, 19].

**Table 1:** Definition of interrater agreement qualitative scores according to Altman et al*.* [15, 16] and Clinical Utility indexes grading according to Mitchell [18]

Agreement classification		Clinical Utility Index classification	
*κ*	Strength of agreement	CU+Ve or CU−Ve	Grading
< 0.21	Poor	< 0.49	Poor utility
0.21–0.40	Fair	0.49–0.63	Satisfactory utility
0.41–0.60	Moderate	0.64–0.80	Good utility
0.61–0.80	Good	0.81–1.00	Excellent utility
0.81–1.00	Very good		

*CU + Ve* positive clinical utility index, *CU−Ve* negative clinical utility index

In all analyses, a two-tailed *p *value < 0.05 was considered significant. GraphPad Prism (San Diego, CA, USA), Stata (version 13.0, College Station, Texas, USA) and R (version 3.6.2) were used for statistical analysis and rendering graphs.

## Results

### Clinical data

**Fig. 4:**
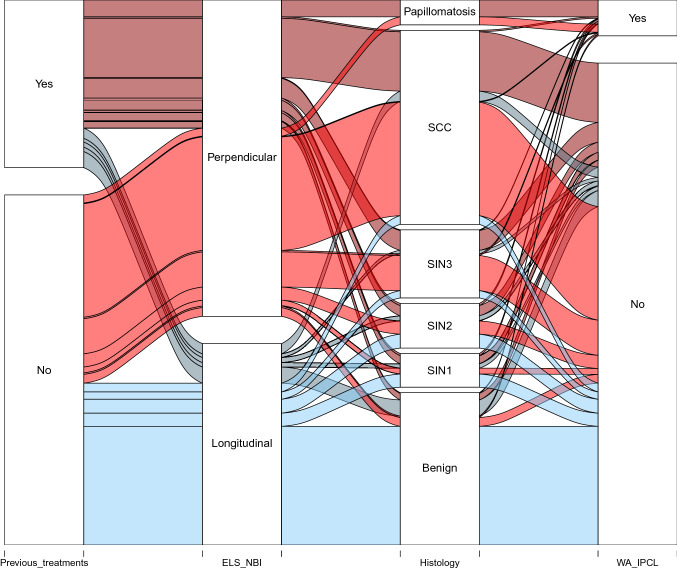
Alluvial chart showing frequency distribution of previous treatment, ELS classification results, histology, and presence of wide angle IPCL features (WA IPCL). Color code according to different matching of previous treatments and ELS classification results

A total of 707 patients met enrolment criteria: 434 (61.3%) had been evaluated and treated at the center A, and 273 (38.7%) at the center B. Five-hundred and fifty six (78.6%) were males and 151 (21.4%) females, with a mean age of 61.8 years (range 18–91). Four-hundred and seventy-eight (67.6%) patients were submitted to endoscopic evaluation and surgical procedures without previous treatments, whereas 174 (24.6%) had been already surgically treated, 17 (2.4%) received head and neck radiotherapy, and 54 (7.6%) had been previously biopsied elsewhere. The final pathologic report was consistent with a benign lesion in 208 (29.5%) cases, papillomatosis without atypia in 34 (4.8%), mild SIN (SIN1) in 46 (6.5%), moderate SIN (SIN2) in 61 (8.6%), severe SIN (SIN3) or CIS in 93 (13.1%), and SCC in 265 (37.5%). Full details are summarized in Table 2, Fig. 4.

**Table 2:** Clinical features of the cohort

Variables	All		Longitudinal vessels		Perpendicular vessels	
	*n*	%	*n*	%	*n*	%
All	707	100.0	283	40.0	424	60.0
Gender						
Male	556	78.6	184	26.0	372	52.6
Female	151	21.4	99	14.0	52	7.4
Previous treatments°						
No	478	67.6	221	31.3	257	36.4
Previous surgery	174	24.6	45	6.4	129	18.2
Previous RT	17	2.4	4	0.6	13	1.8
Previous biopsy	54	7.6	8	1.1	46	6.5
Histology						
Benign	208	29.4	184	26.0	24	3.4
Papillomatosis without atypia	34	4.8	0	0.0	34	4.8
SIN1	46	6.5	24	3.4	22	3.1
SIN2	61	8.6	26	3.7	35	5.0
SIN3/CIS	93	13.2	15	2.1	78	11.0
SCC	265	37.5	26	3.7	239	33.8

The sum of rows is 723 since 16 patients had two different previous treatments

*RT* radiotherapy, *SIN* squamous intraepithelial neoplasia, *CIS* in situ carcinoma, *SCC* squamous cell carcinoma

### ELS classification interobserver reliability

Cohen’s k statistic was used to assess the agreement between judgment of each lesion by two independent raters in each institution applying the ELS classification. According to the criteria by Altman et al. [16, 17], reported in Table 1, for the entire cohort the result was satisfactory and showed a very good agreement between observers at both the center A (*κ* = 0.954; 95% confidence interval [CI] 0.86–1.0, *p* < 0.0001) and the center B (*κ* = 0.872; 95% CI 0.754–0.991, *p* < 0.0001) (Table 3). The agreement was consistent and significant (*p* < 0.0001) for both Institutions, as well as for untreated (*κ* = 0.949; 95% CI 0.833–1.0 and *κ* = 0.864; 95% CI 0.723–1.0, respectively) and previously treated patients (*κ* = 0.945; 95% CI 0.785–1.0 and *κ* = 0.894; 95% CI 0.674–1.0, respectively) (Table 3).

**Table 3:** Agreement analysis by Cohen’s *k* test

Institution	Type of lesion	*N*	%	Agreement (%)	*κ*	95% CI (*κ*)	*p*
University of Genoa (Center A)	All	434	100	97.7	0.954	0.86–1.0	< 0.0001
	Untreated	284	65.4	97.5	0.949	0.833–1.0	< 0.0001
	Previous biopsy/surgery/RT	150	34.6	98.0	0.945	0.785–1.0	< 0.0001
University of Brescia (Center B)	All	273	100	94.9	0.872	0.754–0.991	< 0.0001
	Untreated	194	71.1	94.3	0.864	0.723–1.0	< 0.0001
	Previous biopsy/surgery/RT	79	28.9	96.2	0.894	0.674–1.0	< 0.0001

### Diagnostic performance

**Fig. 5:**
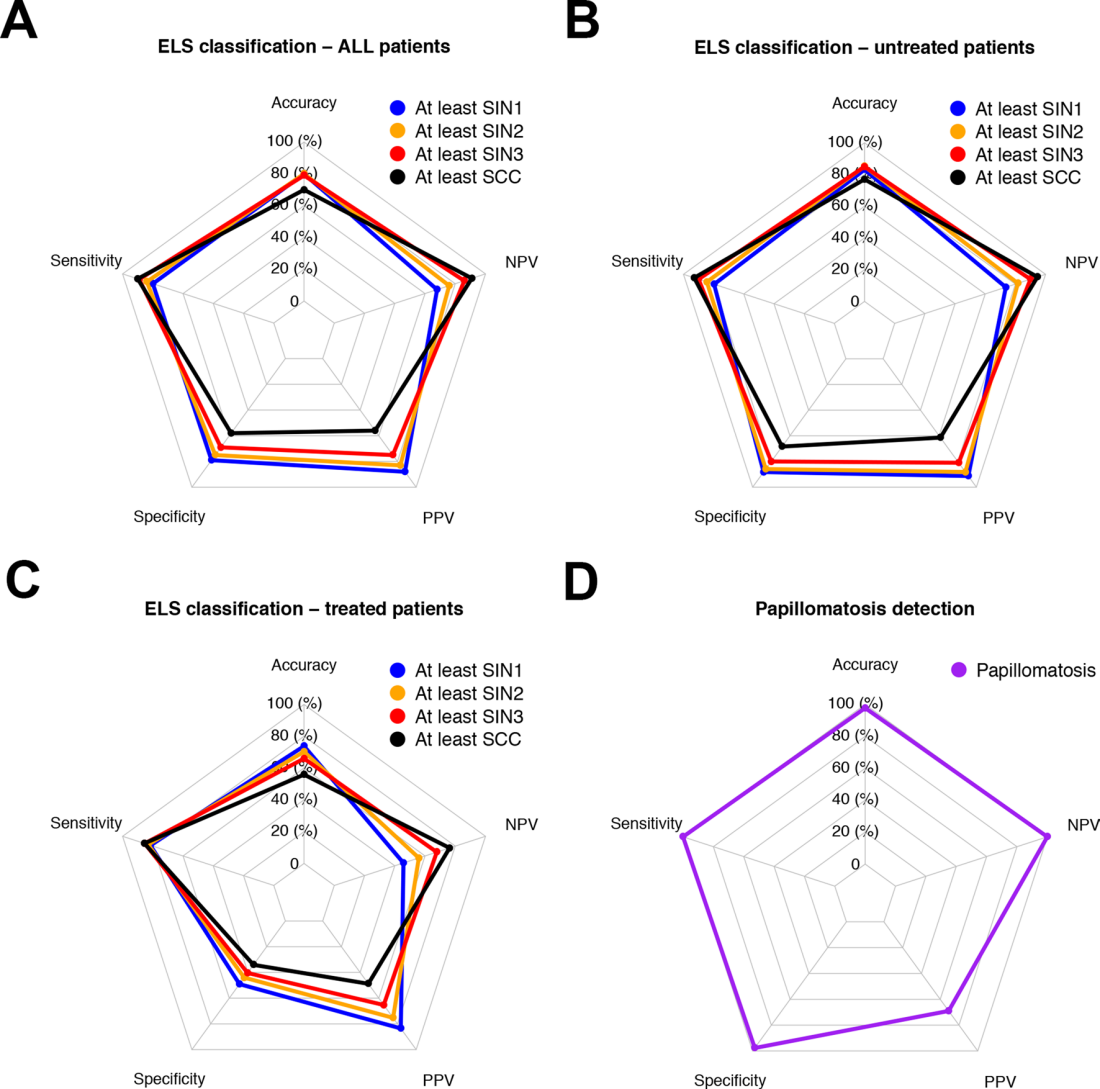
Radar charts showing the diagnostic test applying the ELS classification for the detection of different histologic targets in the whole cohort (**a**), in the untreated group (**b**), and in the previously surgical or RT treated group (**c**). Diagnostic test results referred to the detection of wide angle IPCLs for the diagnosis of laryngeal papillomatosis (**d**)

Considering the final score in the entire cohort (24 cases with initial disagreement were resolved between the examiners), performance of the diagnostic test was assessed investigating the detection of at least SIN1-SCC (Table 4). The best sensitivity and NPV were obtained for detection of SCC (0.90 and 0.91, respectively) and, accordingly, the best specificity and PPV for diagnosis of at least SIN1 (0.79 and 0.88, respectively). Considering previous treatments as a potential source of bias, for untreated patients the ELS classification reached the best performance with sensitivity and NPV for detection of SCC of 0.93 and 0.95, respectively, and specificity and PPV for diagnosis of at least SIN1 of 0.88 and 0.91, respectively. In previously treated patients, the performance of endoscopic evaluation was still satisfactory in terms of sensitivity (from 0.82 to 0.86), while it was poorer in terms of specificity (from 0.34 to 0.49), NPV (from 0.46 to 0.76), and PPV (from 0.45 to 0.84) (Table 4, Fig. 5).

**Table 4:** Diagnostic test results

	Sensitivity (95% CI)	Specificity (95% CI)	PPV (95% CI)	NPV (95% CI)
All				
At least SIN1	0.80 (0.77–0.84)	0.79 (0.74–0.84)	0.88 (0.85–0.91)	0.68 (0.62–0.73)
At least SIN2	0.84 (0.80–0.87)	0.75 (0.70–0.80)	0.83 (0.79–0.87)	0.76 (0.71–0.81)
At least SIN3	0.89 (0.85–0.92)	0.69 (0.64–0.74)	0.75 (0.70–0.79)	0.86 (0.81–0.89)
SCC	0.90 (0.86–0.94)	0.58 (0.53–0.63)	0.56 (0.52–0.61)	0.91 (0.87–0.94)
Untreated				
At least SIN1	0.80 (0.75–0.84)	0.88 (0.83–0.93)	0.91 (0.87–0.95)	0.74 (0.67–0.79)
At least SIN2	0.85 (0.80–0.89)	0.86 (0.81–0.90)	0.88 (0.84–0.92)	0.82 (0.76–0.87)
At least SIN3	0.90 (0.86–0.94)	0.81 (0.75–0.86)	0.81 (0.76–0.86)	0.90 (0.86–0.94)
SCC	0.93 (0.88–0.96)	0.68 (0.63–0.74)	0.61 (0.55–0.67)	0.95 (0.91–0.97)
Previous biopsy/surgery/RT				
At least SIN1	0.82 (0.75–0.87)	0.49 (0.35–0.63)	0.84 (0.77–0.89)	0.46 (0.33–0.59)
At least SIN2	0.83 (0.76–0.89)	0.44 (0.33–0.56)	0.75 (0.68–0.82)	0.56 (0.42–0.69)
At least SIN3	0.85 (0.78–0.91)	0.40 (0.31–0.51)	0.65 (0.58–0.72)	0.68 (0.54–0.79)
SCC	0.86 (0.77–0.92)	0.34 (0.26–0.43)	0.45 (0.41–0.57)	0.76 (0.63–0.86)
Papillomatosis detection	1.00 (0.90–1.00)	0.98 (0.96–0.99)	0.69 (0.55–0.82)	1.00 (0.99–1.00)

*PPV* positive predictive value, *NPV* negative predictive value, *CI* confidence interval, *SIN* squamous intraepithelial neoplasia, *SCC* squamous cell carcinoma

The measurement of the CU indexes confirmed this observation with a good CU + Ve and CU−Ve for all outcomes except one in untreated patients, whereas no more than satisfactory or even poorer results were obtained for most outcomes in previously treated or biopsied patients, as shown in Table 5.

**Table 5:** Clinical Utility indexes and utility grading results according to Mitchell [18]

	CU+Ve (95% CI)	CU+Ve Judgment	CU−Ve (95% CI)	CU−Ve Judgment
All				
At least SIN1	0.71 (0.67–0.75)	Good	0.54 (0.49–0.58)	Satisfactory
At least SIN2	0.70 (0.65–0.74)	Good	0.57 (0.53–0.62)	Satisfactory
At least SIN3	0.66 (0.61–0.71)	Good	0.59 (0.55–0.63)	Satisfactory
SCC	0.51 (0.45–0.57)	Satisfactory	0.53 (0.49–0.57)	Satisfactory
Untreated				
At least SIN1	0.73 (0.68–0.78)	Good	0.65 (0.60–0.70)	Good
At least SIN2	0.75 (0.69–0.80)	Good	0.70 (0.66–0.74)	Good
At least SIN3	0.73 (0.68–0.79)	Good	0.73 (0.69–0.77)	Good
SCC	0.57 (0.50–0.64)	Satisfactory	0.65 (0.61–0.69)	Good
Previous biopsy/surgery/RT				
At least SIN1	0.68 (0.61–0.75)	Good	0.23 (0.11–0.34)	Poor
At least SIN2	0.63 (0.55–0.70)	Satisfactory	0.25 (0.14–0.35)	Poor
At least SIN3	0.56 (0.47–0.64)	Satisfactory	0.27 (0.17–0.38)	Poor
SCC	0.42 (0.32–0.52)	Poor	0.26 (0.16–0.36)	Poor
Papillomatosis detection	0.69 (0.56–0.83)	Good	0.98 (0.97–0.99)	Excellent

*CU + Ve* positive clinical utility index, *CU−Ve* negative clinical utility index, *CI* confidence interval, *SIN* squamous intraepithelial neoplasia, *SCC* squamous cell carcinoma

### Diagnostic performance in respiratory papillomatosis

Among perpendicular vascular changes, the ELS classification well defines the vascular pattern of recurrent respiratory papillomatosis lesions, characterized by vessel loops with wide angle turning point, embedded in a three-dimensional warty structure [15]. We tested the identification of these features by NBI in our cohort, confirming their value for correct identification of this disease with a sensitivity of 1.0 (95% CI 0.90–1.0), specificity of 0.98 (95% CI 0.96–0.99), PPV of 0.69 (95% CI 0.55–0.82), and NPV of 1.0 (95% CI 0.99–1.0), as shown in Table 4. Moreover, the measure of CU indexes confirmed the excellent performance of NBI with a CU–Ve of 0.98 (95% CI 0.97–0.99) and good performance in terms of CU + Ve of 0.69 (95% CI 0.56–0.83), as reported in Table 5.

## Discussion

Among the several bioendoscopic techniques now available for routine evaluation of the UADT, NBI appears to be the most effective in evaluation of the larynx, hypopharynx, oral and oropharyngeal cavities [1, 20, 21]. The easy use of NBI and other bioendoscopic tools based on similar principles, which aims to enhance the vascular features of tissues (e.g*.* SPIES [22] or iSCAN [20]), is mainly due to full integration of high-definition videoendoscopes, easily activated by pressing a button during in-office endoscopic examination or during pre- and intraoperative assessment. Interestingly, the superior in-depth evaluation of the bioendoscopic features of a given lesion may pave the way to the proof of concept of the optical biopsy, i.e*.* the capability to understand the nature of a given vocal fold mucosal lesion before its removal, thus modulating its excisional biopsy and optimizing hospitalization time, costs, and undue damage to surrounding healthy structures [23].

The need for a common language to categorize and share the findings from NBI evaluation led to a number of different classification systems. The first to have widespread diffusion in the head and neck scientific community was proposed by Ni et al*.* [11]. These authors divided the different IPCL changes in five types (I–V), judging them as benign (from types I–IV), suspected malignant, and frankly malignant (type V). However, apart from its intrinsic complexity, this classification clearly showed a lack of a clear-cut threshold between benign and malignant diseases. In fact, different authors proposed different cut-offs for the worst endoscopic feature of each lesion to be considered suspicious, ranging from type III [24], to type IV [25–27], and type V [9, 11, 28].

Therefore, in 2016 the ELS proposed a new classification system for the interpretation of glottic vascular abnormalities detected during NBI-guided endoscopies [15]. This classification considers vascular abnormalities as IPCL perpendicular to the epithelium surface as suspicious, whereas longitudinal vascular changes (e.g*.* dilated or tortuous vessels, increased vessels numbers) are considered as not suspicious to harbor respiratory papillomatosis, pre-malignant, or cancerous lesions. The first attempt to apply this dichotomic classification was in the study by Šifrer et al*.* [29] who analyzed 80 vocal cords lesions in which the identification of a perpendicular vascular pattern was diagnostic for CIS-SCC with a sensitivity of 100%, specificity of 95%, PPV of 88%, and NPV of 100%. Further analysis evaluating a larger cohort of 288 vocal cords gave similar results (sensitivity 98%, specificity 95%, PPV 88%, and NPV 99%) [30].

Our results, obtained in two of the European pioneer centers applying NBI for evaluation of the UADT since 2007, herein confirm the intrinsic value of the ELS classification for laryngeal vascular changes in the identification of lesions harboring pre-cancerous or frankly neoplastic alterations. In particular, we applied this diagnostic tool to demonstrate its possible role in performing a so-called optical biopsy. In fact, our policy has always been, for early glottic lesions, a one-stage modulated excisional biopsy based on a number of pre- and intraoperative diagnostic tests in which WL and NBI rigid endoscopy under general anesthesia has always played a paramount role [23]. Moreover, as asserted by many authors, NBI is capable of enhancing small lesions that are undetectable by WL alone, thus ameliorating the treatment of laryngeal SSC, as well as assessing the potential multifocality of the disease and correct evaluation of intraoperative margins [31], as well as early identification of small recurrences during follow-up that may still allow application of minimally invasive treatments such as laser office-based procedures or second-look microlaryngoscopic operations [32–35]. Of note, the present study demonstrated a lower diagnostic accuracy of NBI in the previously treated patients compared to the untreated ones, thus confirming the potential confounding factor played by invasive sampling procedures when not directed to the full removal (excisional biopsy) of the entire visible lesion within safe margins.

The excellent interobserver reliability of the ELS classification with a *k* > 0.81 in all scenarios tested and reproducible in two independent centers confirms the reproducibility of the operators’ findings in applying this classification tool. The high interobserver reliability of the ELS classification can be explained by its intrinsic simple application and dichotomic arrangement, providing better performance compared to other proposed classification systems such as that by Ni, which is complicated by a 5-tier structure and associated with moderate/substantial interrater accordance, with a *k* ranging from 0.55 to 0.69 [36, 37].

On the other hand, it has to be noted that all the observers involved in this study had a minimum experience of 3 years in the use of NBI technology. Even though application of the ELS Classification on vocal fold vascular changes as observed by NBI is no more subjective than any other diagnostic performance, evaluation of certain subtle and sometimes ambiguous neoangiogenic patterns still may require a higher level of expertise, for which a learning curve is inevitably necessary. However, data derived from the gastrointestinal field show that less than a year of training evaluating 200 cases is enough to guarantee an accurate evaluation of NBI frames and that the motivation of the trainer itself can significantly improve the overall performance [38].

Investigating the diagnostic test, having as a target all the possible grades of pre-malignant or malignant transformation, allowed us to depict the capability of the ELS classification in helping to correctly identify pre-malignant cases with the highest PPV and specificity for at least SIN1 diagnosis. The lower performance of such parameters observed for the final diagnosis of glottic SCC can be explained by the presence, and progressively increase, of perpendicular vascular changes at early stages of pre-malignant transformation (SIN1-SIN2). By contrast, for diagnosis of laryngeal SCC, the ELS classification had good performance in terms of sensitivity and NPV, with a low rate of false negative cases and good confidence in a negative result (absence of perpendicular vascular changes).

Furthermore, several authors have underlined the utility of NBI for detection of recurrent respiratory papillomatosis and its ability to increase the detection rate of small lesions that invisible by WL alone [29, 33, 39–42]. The excellent performance in terms of CU + Ve and CU−Ve searching for wide angle IPCLs in the identification of respiratory papillomatosis mandates, as previously suggested by the recent literature [33, 39–44], the use of biologic endoscopy tools like NBI, and should be considered the endoscopic gold standard for optical biopsy and follow-up of patients affected by laryngeal papillomas.

The main limits of our study are represented by its retrospective design, balanced by analyzing a broad bicentric cohort. Nevertheless, among the estimator analyzed, the suboptimal performance in terms of specificity, negative predictive value, and CU−Ve could has been underestimated having chosen among the inclusive criteria the need for a histopathological diagnosis: several patients without any suspicious lesion at the first evaluation and along time could be considered as true negatives too, thus improving the values of such estimators.

Further developments in this field might include the analysis of a prospective cohort of patients, implementing the enrollment of true negative cases and developing a real-time software applicable in the head and neck, based on artificial intelligence algorithms already tested on retrospective studies [45, 46], thus improving the objectivity and detection rate of these diagnostic tools, as already devised for gastrointestinal tract tumors [47, 48].

## Conclusion

The ELS classification for NBI vascular changes of laryngeal lesions, herein validated in a large multicenter cohort, is a highly reliable tool with good diagnostic performance in the optical biopsy setting, confirming its overall value. The systematic use of this classification seems to allow better (and purely endoscopic) diagnostic capability of suspicious glottic lesions, reliably distinguishing benign ones from those with a diagnosis of papillomatosis, SIN, or invasive SCC. The excellent performance of NBI for correct identification of respiratory papillomatosis also confirms its usefulness in this clinical setting.

## Data Availability

Full dataset will be available at: “ELS_NBI_Classification_Validation_Dataset”, Mendeley Data, V1, https://doi.org/10.17632/txzzw9n7xs.1https://doi.org/10.17632/txzzw9n7xs.1 (Embargo date: 6th December 2021).
